# Geographic Access to Urodynamics Studies in the United States: A Medicare-Based Geospatial Analysis

**DOI:** 10.7759/cureus.114742

**Published:** 2026-08-18

**Authors:** Chetan Giduturi, Sofie Rosenberg, Tyler Muffly

**Affiliations:** 1 Obstetrics and Gynecology, University of Colorado Anschutz Medical Campus, Aurora, USA; 2 Department of Obstetrics and Gynecology, Denver Health Medical Center, Denver, USA

**Keywords:** advanced diagnostic testing, clinical urodynamics, geographic disparity, geospatial analysis, lower urinary tract symptoms (luts), medicare data, obstetrics and gyencology, urogynecology

## Abstract

Objectives: This study aimed to evaluate geographic access to traditional versus video urodynamics testing in the United States and Puerto Rico utilizing Medicare-billing data.

Methods: We performed a national geospatial analysis using the 2024 Medicare Physician and Other Practitioners Public Use File. Clinicians in urology and obstetrics and gynecology who billed Medicare Part B for urodynamics testing were identified using prespecified Healthcare Common Procedure Coding System (HCPCS) codes. Billing addresses from the mainland U.S. and Puerto Rico were cleaned, deduplicated, geocoded, and classified as centers offering traditional or video-associated urodynamics testing. State-level center density was calculated per million women using 2019-2023 American Community Survey population estimates. Centers were classified as metropolitan or nonmetropolitan using 2021 Census core-based statistical area boundaries. Census tract centroids were used as population origins, and distance to the nearest testing center was calculated and female population-weighted nationally and by state.

Results: Approximately 26.2 million women (15.7%) lived more than 100 miles from a video-associated urodynamics testing center, and 800,000 women (0.5%) lived more than 100 miles from a traditional urodynamics testing center. We identified 165 centers billing for video-associated urodynamics testing and 2,397 centers billing for traditional urodynamics testing in the United States and Puerto Rico. Testing centers were concentrated in the Northeast and Florida, with fewer centers across the Great Plains and Mountain West. Among 392 metropolitan areas, 310 (79%) lacked a video-associated urodynamics testing center, and 70 (17.9%) lacked a traditional urodynamics testing center. National median population-weighted distance was 27.8 miles for video-associated urodynamics testing and 3.6 miles for traditional urodynamics testing. The greatest access gaps differed by testing type but generally were observed in rural or low-density states.

Conclusions: Most women in the United States live within a short distance of a center billing for urodynamics testing, but meaningful geographic access gaps remain, particularly in rural regions, the Great Plains, and the Mountain West. These findings highlight the need for regional referral pathways, outreach models, and improved triage systems for patients with complex lower urinary tract symptoms (LUTS) who may require urodynamics evaluation.

## Introduction

Lower urinary tract symptoms (LUTS) are common worldwide and represent a substantial source of morbidity among women [1-3]. These symptoms encompass a broad range of storage, voiding, and post-micturition complaints, including urinary urgency, frequency, nocturia, urinary incontinence, hesitancy, incomplete bladder emptying, and urinary retention [4]. Contemporary population-based studies demonstrate that LUTS affect women across the lifespan, with prevalence generally increasing with age [1-3]. Although LUTS occur in both women and men, several conditions, particularly urinary incontinence, disproportionately affect women because of differences in pelvic anatomy and the effects of pregnancy, childbirth, menopause, aging, and prior pelvic surgery [5-7]. Beyond their physical manifestations, LUTS may negatively affect sleep, mobility, sexual function, social participation, emotional well-being, and overall quality of life [8,9]. The high prevalence and chronic nature of these symptoms create a substantial need for accurate diagnosis and timely access to effective treatment. 

Many women with uncomplicated LUTS can be evaluated using a detailed clinical history, physical examination, urinalysis, bladder diary, postvoid residual measurement, and other basic office assessments. For example, uncomplicated and demonstrable stress urinary incontinence can often be diagnosed without invasive testing, allowing patients to proceed efficiently with conservative or surgical treatment [10]. However, symptoms reported by patients do not always correspond to a single underlying lower urinary tract disorder. Urgency may result from detrusor overactivity, impaired bladder compliance, urinary tract infection, excessive fluid intake, or heightened bladder sensation [11]. Difficulty emptying may reflect bladder outlet obstruction, impaired detrusor contractility, dysfunctional voiding, or a combination of these conditions [12]. Similarly, women with mixed urinary incontinence may have varying contributions from stress incontinence, urgency urinary incontinence, and other abnormalities that cannot always be distinguished through symptoms alone [2]. Diagnostic uncertainty is particularly common among patients with prior incontinence or prolapse surgery, neurologic disease, pelvic organ prolapse, urinary retention, recurrent symptoms after treatment, or discordance between symptoms and examination findings [2]. 

Urodynamic studies (UDS) provide objective information about lower urinary tract function during bladder filling and emptying and are an established component of the evaluation of complex LUTS [13]. Traditional multichannel urodynamic testing generally includes uroflowmetry, filling cystometry, pressure-flow studies, and assessment of pelvic floor or urethral sphincter activity using electromyography when indicated [13,14]. Video urodynamics combine pressure-based urodynamic measurements with fluoroscopic or other contrast-based imaging of the bladder and lower urinary tract [15]. This additional anatomic information may be particularly valuable in selected patients with suspected bladder outlet obstruction, neurogenic lower urinary tract dysfunction, vesicoureteral reflux, abnormal bladder or urethral anatomy, or complex postoperative dysfunction. However, video urodynamic testing requires specialized equipment, trained personnel, access to radiographic imaging, and expertise in interpretation [13,14]. Even traditional multichannel UDS requires equipment, appropriately trained clinicians or technicians, and clinical infrastructure that may not be available in all communities. Consequently, the availability of UDS may depend on referral to a urogynecologist, urologist, or specialized pelvic floor center, potentially requiring patients to travel substantial distances. 

Despite the clinical role of UDS in evaluating complex LUTS, little is known about geographic access to urodynamic testing in the United States. The presence of a clinician or healthcare facility within a region does not necessarily indicate that UDS is offered, that the service is accepting new patients, or that patients can reach the facility within a reasonable travel time. Geospatial studies in other specialties have identified substantial regional disparities in access to services, including oncofertility care, endocrine surgery, and otolaryngology [16-18]. Geographic barriers may delay diagnosis, prolong ineffective treatment, increase the burden of repeated clinical visits, and disproportionately affect patients with limited transportation, inflexible employment, caregiving responsibilities, disability, or financial constraints. These barriers may be particularly important for rural and medically underserved populations, for whom specialty services are often concentrated in distant metropolitan centers. 

The objective of this study was to characterize the geographic distribution and accessibility of urodynamic testing in the United States and to identify areas in which patients may face substantial travel barriers to receiving this service. We hypothesized that access to UDS would vary considerably across regions, with the greatest geographic barriers occurring in rural and less densely populated areas, particularly in the Mountain West and Great Plains. 

## Materials and methods

Geospatial analysis of urodynamics testing was completed utilizing Medicare reimbursement data as a proxy for urodynamics testing usage across the United States. Information on clinicians who billed Medicare Part B for urodynamics testing in CY2024 was obtained from the Centers for Medicare and Medicaid Services (CMS) Medicare Physician and Other Practitioners Public Use file [19]. Data were filtered using the Healthcare Common Procedure Coding System (HCPCS) codes, which cover common urodynamics tests [20]. Codes for video versus traditional urodynamics testing were identified and confirmed by a practicing urogynecologist (T.M.M.). Codes were then downloaded into separate groups and analyzed, respectively. Traditional urodynamics codes include uroflowmetry, cystometry/complex cystometrogram, pressure-flow, EMG, and voiding pressure codes (Table 1). Video urodynamics codes include any dynamic imaging of the lower urinary tract, typically involving fluoroscopy/contrast (Table 2). 

**Table 1:** Traditional urodynamics Healthcare Common Procedure Coding System (HCPCS) codes. HCPCS codes are separated by procedures. Brief descriptions of the procedures are provided.

Traditional HCPCS codes	Code description
51736	Uroflowmetry
51741	Uroflowmetry
51725	Cystometry to measure bladder capacity/pressure
51726	Cystometry to measure bladder capacity/pressure
51727	Complex cystometrogram of urethra
51728	Complex cystometrogram of bladder
51729	Complex measurement of pressure of urine flow in bladder with urethra pressure and voiding pressure studies
51785	Needle measurement and recording of electrical activity of muscles at bladder and bowel openings
51792	Measurement of the electrical activity in the anal or urethral sphincter muscles.
51797	Measurement of voiding pressure from detrusor muscle

**Table 2:** Video urodynamics Healthcare Common Procedure Coding System (HCPCS) codes. HCPCS codes are separated by procedures. Brief descriptions of the procedures are provided. Of note, no Medicare data are available for HCPCS code 51605.

Video HCPCS codes	Code description
51600	Voiding cystourethrogram (VCUG)
		51605	Insertion of a chain into the urethra and/or the bladder during the injection of contrast material for urethrocystography
		51610	Injection of contrast material into the urethra for urethrocystography
74455	Reading voiding cystourethrogram
74430	Reading static cystourethrogram
78740	Injection of contrast to assess ureteral reflux

Filtered data were downloaded as a CSV file and subsequently analyzed in R version 2026.04.0 (R Foundation for Statistical Computing, Vienna, Austria) [21]. Location data for each clinician were cleaned to remove abbreviations and standardize names. Centers were defined as unique addresses, including primary and secondary addresses (such as suite numbers), for each clinician billing for the above procedures. Addresses were deduplicated, including secondary addresses, such as suite numbers, to identify individual centers providing urodynamics testing. To maximize the number of data points from urodynamics testing, only Urology and Obstetrics and Gynecology clinicians were included by filtering in the variable rndrng_prvdr_type. In addition, only states in the mainland U.S. and Puerto Rico were included. Using the tidygeocoder package [22], each unique address was geocoded through Arc Geographic Information System (ArcGIS). State-level counts of urodynamics centers were calculated and visualized. 

State-level density was also calculated utilizing the five-year American Community Survey Data (2019-2023) [23]. Density was calculated as centers per million women in each state. Centers were also classified as metro versus non-metro areas based on the 2021 Topologically Integrated Geographic Encoding and Referencing/Line Core Based Statistical Area (TIGER/CBSA) census tract geobase [24]. 

Using the 2021 TIGER/Line CBSA polygons [25] (as opposed to PIN or zip-code origin), the distance from patients to the nearest center was calculated. Census tract centroids were used as patient origin. Great circle (Albers equal-area, EPSG:5070) was utilized for distance calculations. Distances were population-weighted at the national and state levels using weight functions built into R (wtd.quantile and ggplot2). Centers by state and weighted distance to centers were mapped onto US maps. All data were separated into video-based and traditional urodynamics testing. A full summary of analysis code can be found in Supplemental Materials. No permissions were required to access and utilize any of the previously mentioned databases. 

## Results

**Figure 1:**
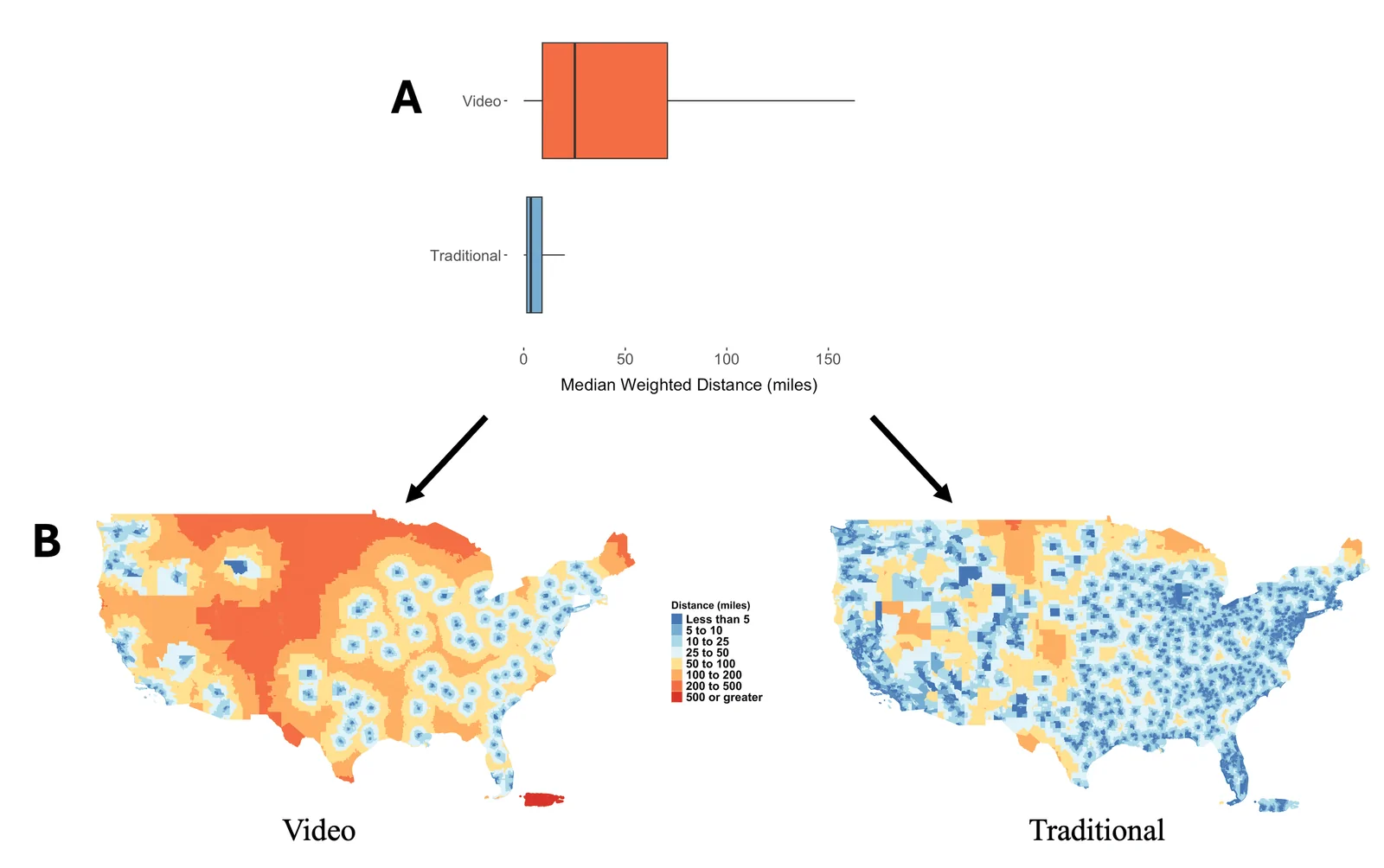
Median weighted distance to urodynamics testing centers. (A) Boxplots of the median weighted distance required to travel for video and traditional urodynamics testing centers. (B) Choropleth maps of the median weighted distance required to travel. Colder colors indicate less distance needed to travel, while warmer colors indicate greater distance required. White/non-colored areas indicate >500 miles required to travel. Figure created by the authors with R (R Foundation for Statistical Computing, Vienna, Austria)

An estimated 26.2 million (15.7%) women are more than 100 miles from any video urodynamics testing center, and 800,000 (0.5%) are more than 100 miles from a traditional urodynamics testing center. The largest median weighted distance was found to be 1,093.9 miles from a video urodynamics testing center and 224.1 miles from a traditional urodynamics testing center (Figure 1). The majority of video urodynamics data fall between 0 and 163.5 miles, with 6,193 (7.4%) outliers, and the majority of traditional urodynamics data fall between 0 and 20.25 miles, with 11,898 (14.2%) outliers (Figure 1A). A majority of video urodynamics outliers are from Puerto Rico populations, but a mixture was present in traditional urodynamics testing data. The median distance by state was also calculated (Table 3). A full list of states and distances can be found in the Appendix. The Mountain West and Great Plains regions appear to have the largest distance/travel burden for U.S. women, with larger distances for video as compared to traditional urodynamics testing. 

**Table 3:** Top 10 states for median weighted distance to urodynamics testing centers.

Video urodynamics		Traditional urodynamics	
States	Distance (miles)	States	Distance (miles)
Puerto Rico	1,044	South Dakota	43
North Dakota	357	Wyoming	40
Colorado	352	Maine	19
Utah	296	Vermont	19
New Mexico	249	North Dakota	13
South Dakota	212	Arkansas	12
Montana	204	Kentucky	11
Wyoming	181	Mississippi	10
Oklahoma	125	New Mexico	10
Arkansas	117	West Virginia	10

**Figure 2:**
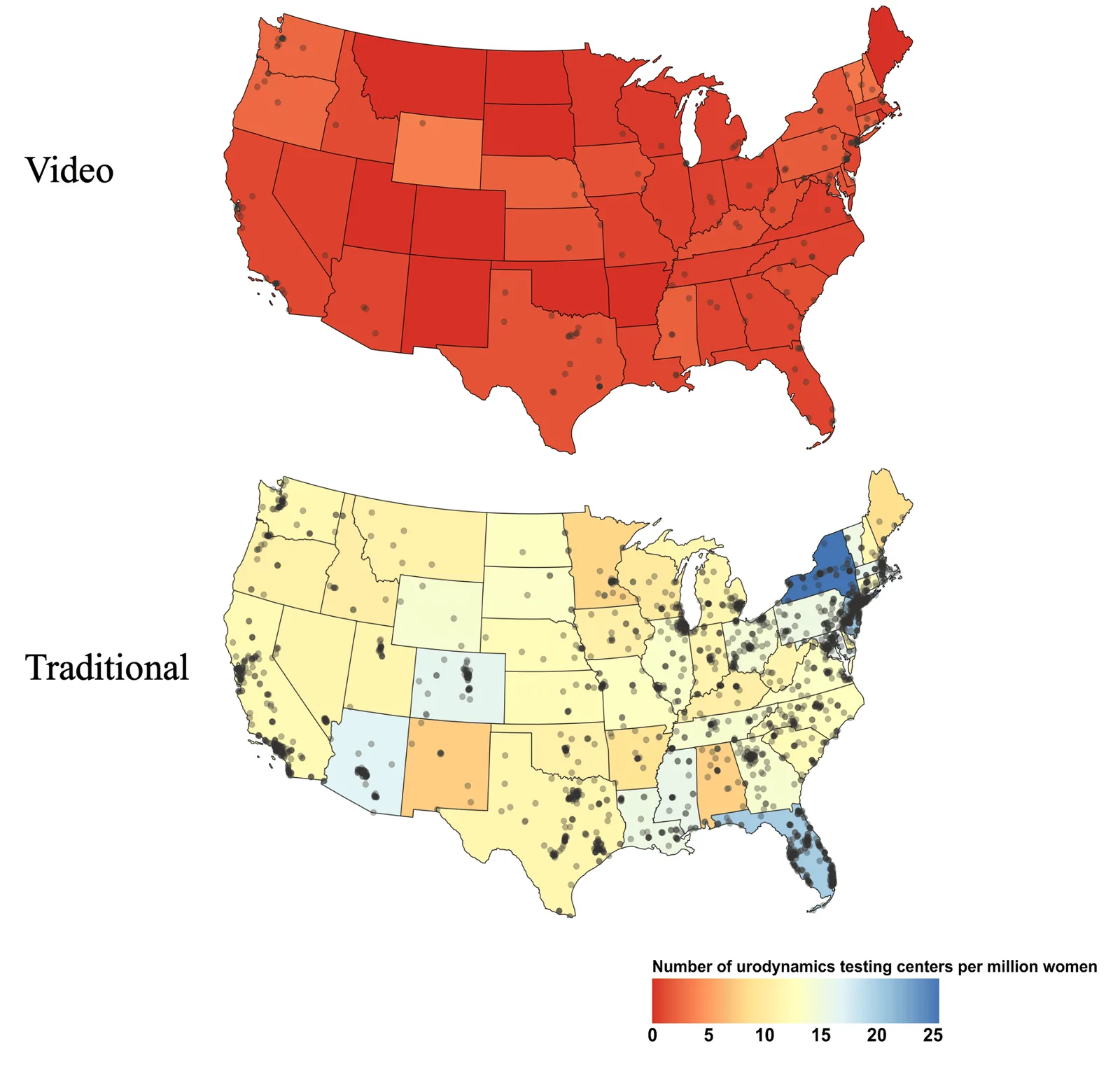
Choropleth map of urodynamics testing centers per million women. Black dots represent testing centers. States are colored by number of urodynamics centers per million women. Includes mainland US and Puerto Rico. Note higher concentrations in the Eastern United States overall compared to the Central and Western regions. A) Video urodynamics testing, B) Traditional urodynamics testing. Figure created by the authors with R (R Foundation for Statistical Computing, Vienna, Austria)

A total of 2,562 urodynamics testing centers were identified in the mainland United States and Puerto Rico. There were 165 video urodynamics centers, with women living a median distance of 27.8 miles from a video testing center, and 2,397 traditional urodynamics testing centers, with a median distance of 3.6 miles. While there are significantly fewer video testing centers compared to traditional ones, they appear to follow a similar distribution pattern. Centers appear to be concentrated in the Northeastern region and Florida, with more traditional testing centers than video testing centers (Figure 2). Generally, the Midwest and Mountain West regions appear to have sparser testing centers. Of 392 metro areas identified, 310 (79%) did not contain a video urodynamics testing center, and 70 (17.9%) did not contain a traditional urodynamics testing center. However, every state or region contains a traditional urodynamics testing center. 

## Discussion

Principal findings

Our analysis identifies geographic barriers in access to both video and traditional urodynamic testing. This includes both availability of testing centers and the distance required to travel to these locations. Our analysis reveals that a majority of U.S. women must travel up to 20 miles to reach a center offering traditional urodynamics testing. While approximately 14% of women travel more than 20 miles, this still represents a significant population that requires longer distances to travel. If requiring video urodynamic testing, individuals may be forced to drive up to 500 miles in the Mountain West and Great Plains region to obtain the appropriate testing. While specific rural regional data were not utilized in this analysis, the non-metropolitan areas contained these rural areas. These findings align with previous research indicating that rural areas tend to have less access to specialty care services [26]. The Mountain West and Great Plains regions appear to have a relative lack of testing centers compared to other regions in the U.S., representing geographical barriers to accessing this care in rural communities. The higher number of metropolitan areas with urodynamics testing may be related to the concentration of technology in metropolitan areas, possibly at academic centers, though further exploration is required. While it is unclear exactly why this is the case, it is consistent with the geospatial access patterns of other nonemergent specialty services, such as otolaryngology or cardiovascular imaging [18,27].

Clinical meaning

While barriers exist to accessing this advanced diagnostic testing, additional research is warranted to differentiate between needs for video vs. traditional urodynamic testing. As previously mentioned, not every patient with incontinence requires urodynamics testing [10]; of these women, not all require video urodynamics, as it may not change management or outcomes [15]. Based on the American Urological Association (AUA)/Society of Urodynamics, Female Pelvic Medicine & Urogenital Reconstruction (SUFU) guidelines on urodynamics testing, evidence for the use of video urodynamics for neurogenic symptoms and bladder neck obstruction is Grade C and Expert Opinion, respectively [15]. While there is likely a role for video urodynamics in patient management, these ratings indicate that more research is needed to define that role. Traditional urodynamics testing is likely sufficient for the diagnosis and management of most women who need testing. Future research on geospatial barriers should integrate actual diagnoses, quality-of-life measures, and outcomes to elucidate factors between video and traditional UDS. 

Policy/practice implications

Overall, these findings indicate a need to improve access to advanced diagnostic testing across the United States. Through the use of telemedicine and outreach clinics, hopefully some of these barriers can be addressed. Tested methods have included telemedicine to help coordinate care before testing to decrease the travel burden to specialty care settings [28]. It has been successfully used for consultations with female pelvic medicine and reconstructive surgery, although in-person visits would still be required for actual testing [29]. In addition, telemedicine requires patients to have access to broadband Internet, which can limit access to telemedicine care in communities without access to specialty care services [30]. Outreach clinics can also improve access for individuals with longer travel times, bringing health professionals closer to women in more rural areas. While beneficial for decreasing the distance barrier, intermittent availability, scheduling, and cost structures may lessen the benefits of this care model [31-33]. While not explored in this study, discussions on improving access in any format will always require an understanding of resource availability, in this case, availability of providers in urology or female pelvic medicine and reconstructive surgery (FPMRS). Average appointment wait times for a provider in FPMRS are around 23 days, with longer wait times for female providers than for male providers [34]. In addition, an estimated one in five academic centers do not have access to an FPMRS provider [35]. Regional referral networks, allowing health systems to prioritize and consolidate referrals for certain specialties, can potentially streamline the referral process to help patients access FPMRS providers [36]. However, Medicare referral networks appear to be fragmented for women's health specialties, such as pelvic floor physical therapy, with gaps in the Central U.S., mirroring the findings present in our study [37]. Including these solutions is crucial to discussions around improving geographical access to urodynamic studies and advanced diagnostic testing. 

Limitations

The limitations of this study include its reliance on Medicare data only. It does not account for urodynamics testing centers accepting other forms of payment, including private insurance, self-pay, and Medicaid. While this study attempts to delineate urodynamics testing centers based on the inclusion of only Urology and OBGYN practices, we were unable to separate male- and female-associated testing. This sorting also does not remove the possibility that other specialties, such as radiology, billed under Urology and OBGYN. In addition, an analysis of access was done in terms of the net distance required to travel. The amount or volume of procedures billed per center was not included. Each center represents a single provider, though it does not reflect patient census, wait times, or other patient demographics. Further analysis should account for actual road-network travel time, terrain types, billing volume, wait times, and costs to create a more robust picture of geographical access to testing centers. Addresses provided in the CMS file could potentially represent billing addresses that differ from actual clinic locations, slightly skewing this analysis. While our analysis delineates between metropolitan and non-metropolitan areas, a more specific analysis of rural areas could be conducted using rural census data, along with demographic factors such as race and socioeconomic status. Future research can assess these factors and focus more specifically on how geographic location affects the severity, morbidity, and treatment of diseases requiring urodynamics testing. However, this study, to the authors' knowledge, represents the first steps toward understanding geographic access to urodynamics testing in the United States.

## Conclusions

Most women in the United States live within a short distance of a center billing for traditional urodynamics testing, but meaningful geographic access gaps remain, particularly in rural regions, the Great Plains, and the Mountain West. These barriers may be greater for centers that bill video urodynamics testing. These findings highlight the need for regional referral pathways, outreach models, and improved triage systems for patients with complex lower urinary tract symptoms who may require urodynamics evaluation.

## Appendices

**Table 4:** Full list of states by median distance from urodynamics testing centers.

Video urodynamics		Traditional urodynamics	
State	Distance (miles)	State	Distance (miles)
Puerto Rico	1044	South Dakota	43
North Dakota	357	Wyoming	40
Colorado	352	Maine	19
Utah	296	Vermont	19
New Mexico	249	North Dakota	13
South Dakota	212	Arkansas	12
Montana	204	Kentucky	11
Wyoming	181	Mississippi	10
Oklahoma	125	New Mexico	10
Arkansas	117	West Virginia	10
Tennessee	108	Alabama	9
Idaho	100	New Hampshire	8
Maine	95	Oklahoma	7
Minnesota	79	Idaho	6
Iowa	71	Iowa	6
Wisconsin	70	Kansas	6
Louisiana	64	Minnesota	6
Alabama	57	Missouri	6
Mississippi	48	South Carolina	6
Kentucky	41	Tennessee	6
West Virginia	40	Wisconsin	6
North Carolina	39	Georgia	5
Rhode Island	38	Indiana	5
Indiana	37	Montana	5
Ohio	35	North Carolina	5
Michigan	34	Delaware	4
South Carolina	32	Louisiana	4
Delaware	25	Michigan	4
Georgia	25	Nebraska	4
Illinois	25	Ohio	4
Florida	24	Oregon	4
Vermont	24	Texas	4
Missouri	23	Virginia	4
New Hampshire	22	Washington	4
Virginia	22	Puerto Rico	4
Kansas	21	Illinois	3
Pennsylvania	19	Nevada	3
Arizona	17	Arizona	3
Massachusetts	16	Pennsylvania	3
Texas	15	Utah	3
Washington	13	California	3
California	13	Colorado	3
Maryland	12	Florida	2
Nevada	12	Maryland	2
Oregon	12	Massachusetts	2
Nebraska	10	New Jersey	2
New Jersey	10	Rhode Island	2
New York	7	District of Columbia	1
District of Columbia	4	New York	1
